# Effects of Thinning Intensities on Soil Infiltration and Water Storage Capacity in a Chinese Pine-Oak Mixed Forest

**DOI:** 10.1155/2014/268157

**Published:** 2014-04-23

**Authors:** Lili Chen, Zhiyou Yuan, Hongbo Shao, Dexiang Wang, Xingmin Mu

**Affiliations:** ^1^Institute of Soil and Water Conservation, Chinese Academy of Science and Ministry of Water Resources, Yangling, Shaanxi 712100, China; ^2^Northwest A&F University, Yangling, Shaanxi 712100, China; ^3^Key Laboratory of Coastal Biology & Bioresources Utilization, Yantai Institute of Coastal Zone Research (YIC), Chinese Academy of Sciences (CAS), Yantai 264003, China

## Abstract

Thinning is a crucial practice in the forest ecosystem management. The soil infiltration rate and water storage capacity of pine-oak mixed forest under three different thinning intensity treatments (15%, 30%, and 60%) were studied in Qinling Mountains of China. The thinning operations had a significant influence on soil infiltration rate and water storage capacity. The soil infiltration rate and water storage capacity in different thinning treatments followed the order of control (nonthinning): <60%, <15%, and <30%. It demonstrated that thinning operation with 30% intensity can substantially improve soil infiltration rate and water storage capacity of pine-oak mixed forest in Qinling Mountains. The soil initial infiltration rate, stable infiltration rate, and average infiltration rate in thinning 30% treatment were significantly increased by 21.1%, 104.6%, and 60.9%, compared with the control. The soil maximal water storage capacity and noncapillary water storage capacity in thinning 30% treatment were significantly improved by 20.1% and 34.3% in contrast to the control. The soil infiltration rate and water storage capacity were significantly higher in the surface layer (0~20 cm) than in the deep layers (20~40 cm and 40~60 cm). We found that the soil property was closely related to soil infiltration rate and water storage capacity.

## 1. Introduction

Recently, the shortage of water resources and the deterioration of water quality have already become important issues in the world [1, 2]. The forest ecosystem plays a significant role in water conservation and water quality improvement and the forest soil is a main carrier of water conservation [3, 4]. Additionally, the soil infiltration rate and water storage capacity are important hydrological parameters in reflecting the soil and water conversation function of forest vegetation [5]. Although a lot of research on soil infiltration and storage capacity of different vegetation in different spatial scales has been performed [6–8], few studies have been done on soil infiltration rate and water storage capacity of pine-oak mixed forest in the Qinling Mountains of China.

The Qinling Mountains play an important role in the physical geographical pattern of China since it is a natural boundary between semihumid monsoon climate of warm temperate zone and humid climate of northern sun-tropical zone. Furthermore, the Qinling Mountains have an irreplaceable role in the ecological service function due to their rich natural geographical conditions. Thus, they gain great attention from scientists and government [9]. The pine-oak mixed forest is composed of* Pinus tabulaeformis* and* Quercus aliena *var.* accuteserra*. It belongs to a kind of significant water conservation forest and distributes widely in the middle Qinling Mountains. However, due to the lack of density management these years, the soil quality and stand growth of pine-oak mixed forest gradually degenerate and decline. Inevitably, the soil water conservation function is seriously affected.

Thinning is a crucial practice in the forest ecosystem management [10, 11]. It has significant influence on forest soil, tree growth, and even the whole forest ecosystem [12, 13]. Most research on thinning mainly has been concentrated on its effects on tree growth laws and stand volume [14–16]. In recent years, the effect of thinning on forest soil has gradually become a hot issue [17–21]. A better understanding of thinning effects on forest soil is helpful to provide more strategies for the scientific management of pine-oak mixed forest and the construction of water conservation forestry in the Qinling Mountains. The present study is conducted in the pine-oak mixed forest 3 years after thinning in the Qinling Mountains. The main objectives are (1) to compare the soil infiltration rates and water storage capacities of three different thinning intensities (15%, 30%, and 60%) and (2) to identify the appropriate thinning intensity of pine-oak mixed forest which has the best function of soil water conservation.

## 2. Study Area and Methods

### 2.1. Study Area and Species

**Figure 1:**
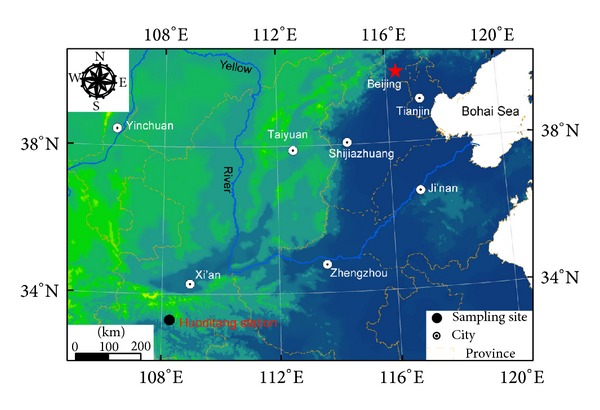
Location of the Huoditang area at Qinling Mountains in Ningshan county of Shaanxi province.

The study was carried out in Huoditang area (33°25′~33°29′N, 108°25′~108°30′E, and 1300–1800 m above the sea) at Qinling Mountains, Ningshan county of Shaanxi province (Figure 1). The climate belongs to the moist mountain climate of warm temperate zone, with an annual mean temperature of 9°C. Mean annual precipitation is 1100 mm with July to September being the wettest period. Mean annual evaporation is 875 mm. Total annual sunshine hours are 1100~1300 h. The soil types in the study area are burozem (Chinese classification) and Hapli-Udic Cambosols under the US soil taxonomy classification system. The pine-oak mixed forest belongs to a kind of typical vegetation type in this area. The dominant arbor species include* Quercus aliena *var.* accuteserra* and* Pinus tabulaeformis*. The dominant shrubs include* Rubus corchorifolius*,* Lespedeza bicolor,* and* Elaeagnus pungens* and the dominant herbs include* Thalictrum aquilegifolium*,* Carex distachya,* and* Agropyron cristatum*.

### 2.2. Plots Setting and Soil Sampling

The pine-oak mixed forest in the study area belongs to natural secondary and middle-aged forest which has been well protected since the 1970s. In May 2009, a fixed plot of approximately 0.5 hm^2^ was set up for the plot investigation in the study area. The thinning intensities in the fixed plot were 15%, 30%, and 60%, and a control (no thinning processing) group as well. Each thinning intensity treatment included 3 standard plots (20 × 20 m^2^). In August 2012, the basic characteristics of standard plots were investigated (Tables 1 and 2). In each standard plot, the soil samples were, respectively, collected from three layers (0~20 cm, 20~40 cm, and 40~60 cm) in each soil profile, and there were 3 soil profiles along a diagonal direction. The collected soil samples were quickly taken back to the lab for testing the soil infiltration rate, soil water storage capacity, and soil properties. Each test was repeated three times.

### 2.3. Determination of Soil Infiltration Rate

Soil infiltration rate was determined using the double cutting ring infiltrometer [22]. The stable infiltration rate was the infiltration amount when it tended to be stable in a unit time. The initial infiltration rate (*V*_*i*_, mm min^−1^) and the average infiltration rate (*V*_*a*_, mm min^−1^) were calculated as follows: (1) *V*_*i*_ = *L*_*i*_/*T*_*i*_; (2) *V*_*a*_ = *L*_*a*_/*T*_*a*_, where *L*_*i*_ (mm) is the infiltration amount in initial infiltration time and *T*_*i*_ (min) is the initial infiltration time. *L*_*a*_ (mm) is the total infiltration amount after reaching stability and *T*_*a*_ (min) is the stable infiltration time.

### 2.4. Calculation of Soil Water Storage Capacity

Soil water storage capacity is an important index to evaluate the soil water conservation ability. The soil maximal water storage capacity (*W*_*t*_, t hm^−2^) and noncapillary water storage capacity (*W*_*a*_, t hm^−2^) were derived from the following equations [23]: (1) *W*_*t*_ = 10 000*P*_*t*_ *h*; (2) *W*_*a*_ = 10 000*P*_*a*_ *h*, where *P*_*t*_ (%) indicates the total porosity of soil, *P*_*a*_ (%) indicates the noncapillary porosity of soil, and *h* (m) indicates the depth of soil layer.

### 2.5. Determination of Soil Physical Properties, Organic Matter, Microbial Quantities, and Enzyme Activities

Soil bulk density and porosity were determined as described by Picchio et al. [11]. Natural water content was measured using incubator (85°C). Soil pH was analyzed by the ZD-2 type potentiometric titration meter. The content of organic matter was determined as described by Nelson and Sommers [24]. Soil microbial quantities were calculated using the dilution plating method [25]. The catalase activity, dehydrogenase activity, urease activity, and invertase activity were measured, respectively, as described by Johnson and Temple [26], Margesin et al. [27], Nannipieri et al. [28], and Frankenberger and Johanson [29].

### 2.6. Statistical Analysis

Experimental data were analyzed using SPSS 18.0 (SPSS Inc., Chicago, IL, USA). One-way analysis of variance (one-way ANOVA) was employed to test the differences in soil infiltration rate and water storage capacity among various soil layers and thinning intensities. Duncan's test (*α* = 0.05) was performed to determine the differences among means within ANOVA. Pearson's correlation analysis was performed to determine the correlation among soil infiltration rates, water storage capacities, and soil factors. Data in tables of this paper are the mean ± standard error.

## 3. Results

### 3.1. Soil Property Changes after Thinning

The soil properties changed dramatically after thinning (Table 3). The lowest soil bulk density (1.17 g cm^−3^) was found in thinning intensity of 30%. In addition, the highest soil total porosity, noncapillary porosity, organic matter content, microbial quantities (bacteria, actinomyces, and fungus), and enzyme activities (catalase, dehydrogenase, urease, and invertase) were also found in thinning intensity of 30%, while the soil pH had no obvious change under different thinning intensities.

### 3.2. Thinning Effects on Soil Infiltration Rate

**Figure 2:**
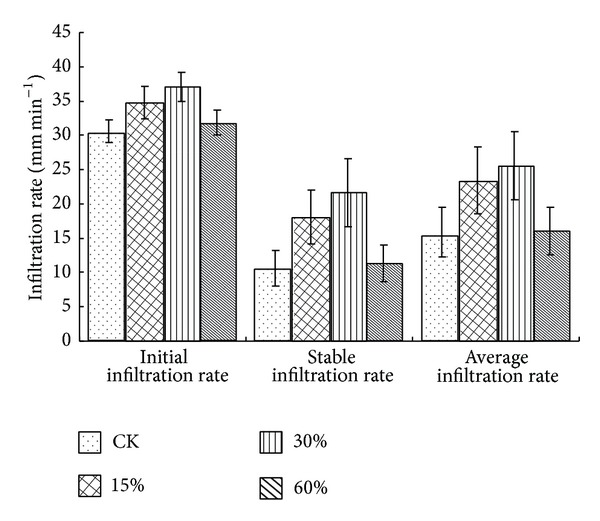
Soil infiltration rates of different thinning intensities.

Appropriate thinning significantly improved the soil infiltration rate. In pine-oak mixed forest, the soil infiltration rate firstly increased with the increase of stand density but then decreased after arriving at a certain extent (Figure 2). The soil initial infiltration rate, stable infiltration rate, and average infiltration rate of different thinning intensities increased in the same order of control (nonthinning): <60% <15% <30%. The soil initial infiltration rate, stable infiltration rate, and average infiltration rate in thinning 30% and 15% were significantly different (*P* < 0.05) from the control but in thinning 60% had no significant difference (*P* > 0.05) from the control. Compared with the control, the soil initial infiltration rate, stable infiltration rate, and average infiltration rate in thinning 30% were, respectively, increased by 21.1%, 104.6%, and 60.9% and in thinning 15% were, respectively, increased by 13.5%, 71.1%, and 47.4%. Additionally, the soil initial infiltration rate, stable infiltration rate, and average infiltration rate from three soil layers were, respectively, in a range of 24.43~44.38 mm min^−1^, 1.76~38.37 mm min^-1,^ and 4.76~42.97 mm min^−1^ and significantly decreased (*P* < 0.01) with the increase of soil depth (Table 4; Figure 2).

### 3.3. Thinning Effects on Soil Water Storage Capacity

**Figure 3:**
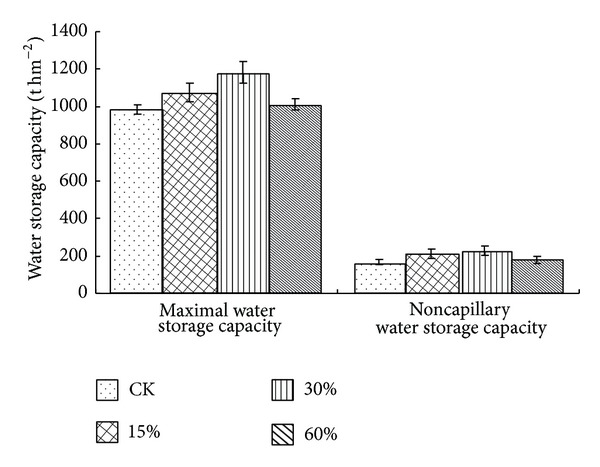
Soil water storage capacities of different thinning intensities.

The soil water storage capacity of pine-oak mixed forest could be increased significantly with appropriate thinning practices. The water storage capacity firstly increased with the increase of stand density but then decreased after reaching a certain degree (Figure 3). The soil maximal water storage capacity and noncapillary water storage capacity of different thinning intensities followed the same order of control (nonthinning): <60% <15% <30%. The soil maximal water storage capacity and noncapillary water storage capacity in thinning 30% and 15% were significantly different (*P* < 0.05) from the control whereas in thinning 60% had no significant difference (*P* > 0.05) from the control. In contrast to the control, the soil maximal water storage capacity and noncapillary water storage capacity in thinning 30% were, respectively, increased by 20.1% and 34.3% and in thinning 15% were, respectively, increased by 9.1% and 26.4%. Besides, both the soil maximal water storage capacity and noncapillary water storage capacity decreased significantly (*P* < 0.01) with the soil depth addition and they were, respectively, in a range of 909.20~1381.13 t hm^−2^ and 117.13~311.67 t hm^−2^ from three soil layers (Table 4).

### 3.4. Relationships among Soil Infiltration Rates, Water Storage Capacities, and Soil Properties under Different Thinning Intensities

The soil physicochemical and biological properties were closely related to the soil infiltration rate and water storage capacity (Table 5). The indexes of soil infiltration rate and water storage capacity were significantly negatively correlated with soil bulk density and significantly positively correlated with soil total porosity, noncapillary porosity, organic matter content, bacteria, actinomyces, fungus, catalase, dehydrogenase, and urease, while the correlation with natural soil water content and soil pH was not significant.

## 4. Discussion

This paper mainly studied the thinning effects on soil infiltration rate and water storage capacity. We found that thinning operations had a significant influence on soil infiltration rate and water storage capacity of pine-oak mixed forest, similar to the observation by Olajuyigbe et al. [30]. These results demonstrate that thinning operation of 30% intensity could substantially improve soil infiltration rate and water storage capacity of pine-oak mixed forest (Figures 2 and 3). The soil infiltration rate and water storage capacity of medium density (1500~1800 trees hm^−2^) forest were significantly higher than those of higher density (2100 trees hm^−2^) forest and lower density (900 trees hm^−2^) forest because the forest of medium density is in a better condition of adequate water, nutrients, and small pressure intraspecific competition to improve the soil infiltration rate and water storage capacity. However, too high or low density would inhibit ecological functions of forest soil, which is similar to the finding of Zhang et al. [31]. Therefore, the soil infiltration rate and water storage capacity could be regulated by adjusting stand density in the forest management.

The thinning operations can influence environmental conditions in forestland such as light, temperature, and soil properties [11, 32]. This study showed that the soil property changed dramatically after thinning (Table 3). We found that the soil property was closely related to soil infiltration rate and water storage capacity (Table 5). The soil bulk density was significantly negatively correlated with the soil infiltration rate and water storage capacity which indicates that soil with smaller bulk density has better infiltration rate and water storage capacity. This result is in substantial agreement with that of Bangita and Rao [33]. Some authors have suggested that the soil infiltration rate and water storage capacity are related to the soil texture, structure, porosity, and organic matter content [5, 34]. We found that the soil infiltration rate and water storage capacity notably increased with the increase of soil total porosity, noncapillary porosity, and organic matter content. This finding is in accordance with previous report [35]. Gao et al. indicate that the natural soil water content is closely related to the soil infiltration rate and water storage capacity [36], but, in this study, we found that the soil infiltration rate and water storage capacity had no significant correlation with natural soil water content. The reason may be that the natural soil water content in this study area is lower than other areas; as a result, it has not shown the impacts on soil initial infiltration value and matric potential gradient volume. The soil biological activity had a significant influence on soil infiltration rate and water storage capacity, consistent with the finding by Rauch-Williams and Drewes [37].

The soil infiltration rate and water storage capacity caused significant difference among different soil layers (Table 4; Figure 2). They were significantly higher in the surface layer (0~20 cm) than in the deep layers (20~40 cm and 40~60 cm). This is because the surface soil is covered by litter with a large quantity of biological return, high organic matter content, rich nutrient, and water [38, 39]. Besides, due to the influence of vegetation roots, the soil structure becomes loose and porous with more organic hydrocolloids, and it can conserve more soil water [40].

Actually, the soil infiltration and water storage capacity are complex hydrological processes. As the soil infiltration rate and water storage capacity are not only affected by the types and structure of the forest, but also closely related to the litter amount, root distribution, and surface runoff [5]. Additionally, the conclusion was preliminary since this study was only based on investigation results of middle-aged pine-oak mixed forest. Thus, further study is needed on pine-oak mixed forest before making comprehensive conclusions.

## 5. Conclusions

Pine-oak mixed forest is an important water conservation forest in Qinling Mountains. As for the forest with stand density higher than 2100 trees hm^−2^, selecting appropriate thinning intensity had an obvious effect on improvement of soil infiltration rate and water storage capacity. In this study, the soil infiltration rate and water storage capacity in thinning 30% were the best, in thinning 15% were the second, and in thinning 60% were the worst. From these results, we conclude that thinning operation with 30% intensity is the optimal practice for improving the soil infiltration rate and water storage capacity of pine-oak mixed forest in Qinling Mountains of China.

## Figures and Tables

**Table 1:** Stand characteristics of the standard plots.

Thinning intensity	Stand density(trees hm^−2^)	Species	Average DBH(cm)	Average height(m)	Canopy density(%)
CK	2100	I/II	24.97	18.50	0.76
15%	1800	I/II	26.87	18.47	0.77
30%	1500	I/II	28.17	18.77	0.76
60%	900	I/II	25.80	18.70	0.75

I: *P. tabulaeformis*; II: *Q. aliena* var. *accuteserra*.

**Table 2:** General status of soil profiles.

Sampling depth (cm)	Color	Texture	Tightness	Humidity
0∼20	Black-brown	Light loam	Loose	Moist
20∼40	Brown	Medium/heavy loam	Moderate	Moist
40∼60	Yellow-brown	Clay	Relatively compact	Slightly moist

**Table 3:** Characteristics of forest soil properties.

Index	Thinning intensity			
	CK	15%	30%	60%
Bulk density (g cm^−3^)	1.24 ± 0.00^a^	1.20 ± 0.01^b^	1.17 ± 0.01^c^	1.23 ± 0.01^a^
Total porosity (%)	49.20 ± 0.80^c^	53.70 ± 0.68^b^	59.11 ± 1.89^a^	50.48 ± 0.57^bc^
Noncapillary porosity (%)	8.47 ± 0.15^b^	10.71 ± 0.02^a^	11.37 ± 0.65^a^	9.06 ± 0.28^b^
Natural water content (%)	5.70 ± 0.08^c^	10.08 ± 0.44^b^	10.63 ± 0.76^b^	12.86 ± 0.39^a^
pH	6.47 ± 0.03^a^	6.53 ± 0.03^a^	6.40 ± 0.12^a^	6.53 ± 0.09^a^
Organic matter (g kg^−1^)	17.11 ± 1.60^b^	22.32 ± 0.49^a^	22.88 ± 0.80^a^	18.12 ± 1.27^b^
Bacteria (10^7^ g^−1^)	22.55 ± 1.31^b^	33.52 ± 1.58^a^	36.91 ± 4.06^a^	24.95 ± 1.72^b^
Actinomyces (10^4^ g^−1^)	25.23 ± 0.58^b^	37.63 ± 0.80^a^	42.59 ± 3.30^a^	28.24 ± 2.15^b^
Fungus (10^3^ g^−1^)	2.59 ± 0.23^b^	4.88 ± 0.17^a^	5.93 ± 0.42^a^	3.25 ± 0.69^b^
Catalase (mL g^−1^)	16.29 ± 2.94^c^	24.18 ± 1.07^ab^	25.99 ± 0.70^a^	19.18 ± 0.08^bc^
Dehydrogenase (*μ*g g^−1^)	115.50 ± 14.04^c^	195.99 ± 3.96^ab^	212.38 ± 9.47^a^	169.52 ± 6.18^b^
Urease (mg g^−1^)	0.14 ± 0.02^b^	0.16 ± 0.01^ab^	0.20 ± 0.01^a^	0.12 ± 0.02^b^
Invertase (mg g^−1^)	17.04 ± 0.70^ab^	17.57 ± 0.68^ab^	19.37 ± 0.69^a^	15.76 ± 0.80^b^

Different small letters in the same row mean significant difference at 0.05 level.

**Table 4:** Characteristics of soil infiltration rate and water storage capacity.

Thinning intensity	Stand density(trees hm^−2^)	Soil layer(cm)	Initial infiltration rate(mm min^−1^)	Stable infiltration rate(mm min^−1^)	Average infiltration rate(mm min^−1^)	Maximal water storage capacity(t hm^−2^)	Noncapillary water storage capacity(t hm^−2^)
CK	2100	0∼20	35.67 ± 1.30^bd^	19.05 ± 1.78^cd^	29.12 ± 2.49^bd^	1061.80 ± 24.75^bd^	211.53 ± 5.87^bd^
		20∼40	31.75 ± 0.52^ce^	10.99 ± 1.73^be^	13.79 ± 2.08^be^	980.93 ± 4.85^be^	179.47 ± 3.32^be^
		40∼60	24.43 ± 0.45^cf^	1.76 ± 0.47^bf^	4.76 ± 0.69^cf^	909.20 ± 21.27^bf^	117.13 ± 10.55^bf^

15%	1800	0∼20	42.82 ± 0.53^ad^	29.73 ± 1.16^bd^	40.67 ± 3.01^ad^	1262.13 ± 20.56^ad^	303.93 ± 5.24^ad^
		20∼40	34.82 ± 0.54^abe^	21.20 ± 1.75^ae^	21.87 ± 1.99^ae^	1021.67 ± 11.74^be^	197.67 ± 1.92^be^
		40∼60	26.54 ± 0.46^bf^	3.50 ± 0.46^abf^	7.73 ± 0.74^abf^	938.20 ± 9.37^bf^	140.80 ± 4.45^abf^

30%	1500	0∼20	44.38 ± 0.70^ad^	38.37 ± 3.04^ad^	42.97 ± 3.05^ad^	1381.13 ± 71.18^ad^	311.67 ± 20.76^ad^
		20∼40	36.74 ± 0.79^ae^	21.95 ± 1.81^ae^	24.66 ± 2.12^ae^	1163.40 ± 32.76^ae^	224.47 ± 12.64^ae^
		40∼60	30.06 ± 0.50^af^	4.77 ± 0.87^af^	9.09 ± 1.05^af^	1002.07 ± 12.63^af^	146.07 ± 6.19^af^

60%	900	0∼20	37.29 ± 0.97^bd^	19.89 ± 2.26^cd^	28.06 ± 2.97^bd^	1116.87 ± 32.30^bd^	243.67 ± 15.52^bd^
		20∼40	33.32 ± 0.45^bce^	11.73 ± 2.56^be^	13.98 ± 2.23^be^	996.47 ± 18.24^be^	180.60 ± 8.30^be^
		40∼60	24.91 ± 0.20^cf^	2.30 ± 0.57^bf^	6.11 ± 0.72^bcf^	915.67 ± 4.55^bf^	119.20 ± 5.06^bf^

Different small letters mean significant difference at 0.05 level; ^abc^mean difference among different densities of the same soil layer; ^def^mean difference among different soil layers of the same density.

**Table 5:** Correlation analysis among soil infiltration rates, water storage capacities, and soil properties.

Soil factor	Initial infiltration rate	Stable infiltration rate	Average infiltration rate	Maximal water storage capacity	Noncapillary water storage capacity
Bulk density	−0.920**	−0.893**	−0.868**	−0.962**	−0.913**
Total porosity	0.931**	0.896**	0.881**	1.000**	0.857**
Noncapillary porosity	0.891**	0.914**	0.821**	0.858**	1.000**
Natural water content	0.355	0.253	0.168	0.291	0.398
pH	−0.227	−0.271	−0.159	−0.201	−0.314
Organic matter	0.846**	0.853**	0.867**	0.842**	0.888**
Bacteria	0.880**	0.813**	0.825**	0.816**	0.685*
Actinomyces	0.925**	0.950**	0.858**	0.840**	0.972**
Fungus	0.918**	0.885**	0.874**	0.917**	0.851**
Catalase	0.884**	0.874**	0.862**	0.843**	0.802**
Dehydrogenase	0.876**	0.842**	0.792**	0.845**	0.891**
Urease	0.728**	0.756**	0.691*	0.854**	0.742**
Invertase	0.587*	0.672*	0.513	0.659*	0.728**

*P < 0.05; **P < 0.01.
